# Supplementary material for: Causal Relationship between Aging and Anorexia Nervosa: A White-Matter-Microstructure-Mediated Mendelian Randomization Analysis
Source: Biomedicines. 2024 Aug 16;12(8):1874. doi: 10.3390/biomedicines12081874 (PMC11351342; doi:10.3390/biomedicines12081874)
Supplement: Supplementary file 1 [file biomedicines-12-01874-s001.zip › Supplementary Figure/Figure S4/Singe SNP plot of MR analysis for TL to ubm-b-1931.pdf]

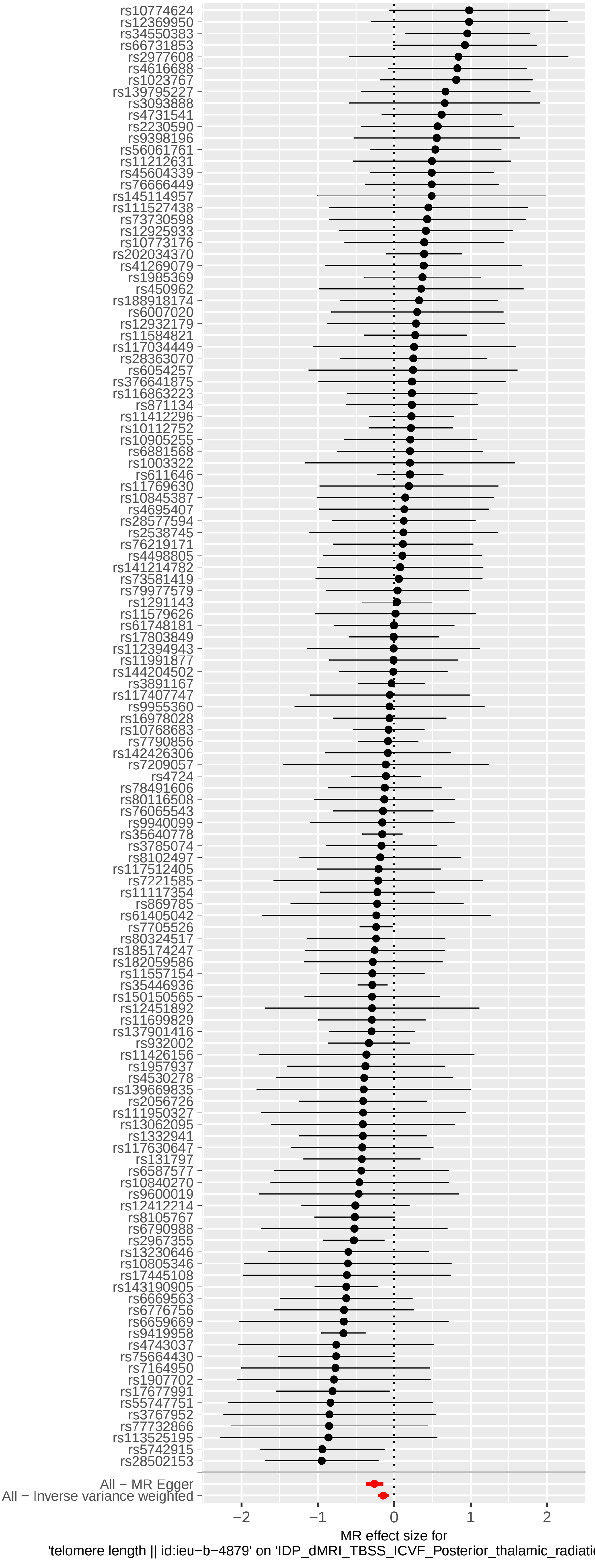
rs10774624
rs12369950
rs34550383
rs66731853
rs2977608
rs4616688
rs1023767
rs139795227
rs3093888
rs4731541
rs2230590
rs9398196
rs56061761
rs11212631
rs45604339
rs76666449
rs145114957
rs111527438
rs73730598
rs12925933
rs10773176
rs202034370
rs41269079
rs1985369
rs450962
rs188918174
rs6007020
rs12932179
rs11584821
rs117034449
rs28363070
rs6054257
rs376641875
rs116863223
rs871134
rs11412296
rs10112752
rs10905255
rs6881568
rs1003322
rs611646
rs11769630
rs10845387
rs4695407
rs28577594
rs2538745
rs76219171
rs4498805
rs141214782
rs73581419
rs79977579
rs1291143
rs11579626
rs61748181
rs17803849
rs112394943
rs11991877
rs144204502
rs3891167
rs117407747
rs9955360
rs16978028
rs10768683
rs7790856
rs142426306
rs7209057
rs4724
rs78491606
rs80116508
rs76065543
rs9940099
rs35640778
rs3785074
rs8102497
rs117512405
rs7221585
rs11117354
rs869785
rs61405042
rs7705526
rs80324517
rs185174247
rs182059586
rs11557154
rs35446936
rs150150565
rs12451892
rs11699829
rs137901416
rs932002
rs11426156
rs1957937
rs4530278
rs139669835
rs2056726
rs111950327
rs13062095
rs1332941
rs117630647
rs131797
rs6587577
rs10840270
rs9600019
rs12412214
rs8105767
rs6790988
rs2967355
rs13230646
rs10805346
rs17445108
rs143190905
rs6669563
rs6776756
rs6659669
rs9419958
rs4743037
rs75664430
rs7164950
rs1907702
rs17677991
rs55747751
rs3767952
rs77732866
rs113525195
rs5742915
rs28502153
All − MR Egger
All − Inverse variance weighted
−2
−1
0
1
2
MR effect size for
'telomere length || id:ieu−b−4879' on 'IDP_dMRI_TBSS_ICVF_Posterior_thalamic_radiati
